# Micro Management: Understanding How Diesel Exhaust Particles Alter Cellular Processes

**DOI:** 10.1289/ehp.117-a504b

**Published:** 2009-11

**Authors:** Cynthia Washam

**Affiliations:** **Cynthia Washam** writes for *EHP, Oncology Times*, and other science and medical publications from South Florida

Scientists have known for decades that people living in cities are more susceptible to certain respiratory diseases than are their countryside counterparts. But they haven’t been able to explain why one urbanite develops severe asthma while his neighbor breathing the same city air has healthy lungs. Now researchers are beginning to solve that riddle as they delve into epigenetics, the emerging science of how environmental factors alter gene expression **[*EHP*** **117:1745–1751; Jardim et al.]**.

This new study focuses on airborne particulate matter, which has long been linked with respiratory disease. Emissions from diesel engines are a prominent source of particulate matter, and diesel exhaust particles (DEP) are classified by the U.S. Environmental Protection Agency as a likely carcinogen. DEP also has been associated with several respiratory disorders including pulmonary inflammation, asthma, and chronic obstructive pulmonary disease.

The authors speculated that pulmonary inflammation due to DEP exposure could be the result of altered microRNA (miRNA) expression, or activation, in cells lining the respiratory tract. MiRNAs are small molecules that regulate gene expression. Studies have connected aberrant miRNA expression with several diseases including cancer, heart disease, neurodegenerative disorders, and congenital organ defects. Few studies, though, have examined whether exposures to environmental contaminants alter miRNA expression.

The authors studied miRNA expression in bronchial epithelial cells, one of the first targets of inhaled particulates. They collected the cells from the airways of healthy, nonsmoking adults and cultured them in a novel air–liquid interface in which differentiated cells were exposed on one side to air, mimicking the environment of the human airway. DEP generated by a diesel automobile engine was suspended in a liquid that was poured on the cell culture.

MiRNA expression changed significantly following DEP exposure. Expression increased in many of the miRNAs and decreased in others. Software identified interrelations between the expression of different miRNAs to assess whether the pattern of up- and down-regulation was consistent with specific biologic pathways. The authors report that pathways involved in inflammation and tumorigenicity are implicated by the patterns they observed.

Earlier studies have shown that DEP prompts the release of several proinflammatory immunoregulatory proteins called cytokines. The authors of the current study suggest this response may be at least partly regulated by changes in the pattern of miRNA expression. The authors believe these alterations may be the first steps toward respiratory disease, and they predict future studies will provide a clearer picture of how expression patterns relate to disease.

